# Racial Disparities in Unmet Pain Treatment Preference, Pain Treatment Satisfaction and Subsequent Opioid Misuse: A Secondary Analysis of a National Multisite RCT

**DOI:** 10.1007/s11606-025-09637-w

**Published:** 2025-06-25

**Authors:** Max Jordan Nguemeni Tiako, Eden Engel-Rebitzer, Ari Friedman, Frances Shofer, Abby Dolan, Erik P. Hess, Jeanmarie Perrone, Marilyn M. Schapira, Zachary F. Meisel

**Affiliations:** 1 Division of General Internal Medicine and Health Services Research, Department of Medicine, David Geffen School of Medicine at UCLA, Los Angeles, CA USA; 2 Department of Medicine, Brigham & Women’s Hospital, Boston, MA USA; 3 Department of Emergency Medicine, Perelman School of Medicine at the University of Pennsylvania, Philadelphia, PA USA; 4 Department of Emergency Medicine, Vanderbilt University Medical Center, Nashville, TN USA; 5 Division of General Medicine, Department of Medicine, Perelman School of Medicine at the University of Pennsylvania, Philadelphia, PA USA

**Keywords:** opioid, unmet preference, opioid misuse, emergency department, acute pain, opioid overdose epidemic, racial disparities, COMM

## Abstract

**Background:**

Racial disparities in opioid prescriptions for pain are well documented. Evidence shows undertreated acute pain increases the risk of developing chronic pain, which puts patients at risk of long-term opioid use and misuse. We sought to determine the association between satisfaction with analgesia, unmet opioid preference, and opioid misuse risk by race in a diverse, longitudinal cohort.

**Methods:**

We conducted a secondary analysis of participants with complete data in an RCT of 1301 patients who presented to the emergency department (E.D.) for acute kidney or back pain. Our primary outcome was opioid misuse risk quantified by the current opioid misuse measure (COMM), a self-report 17-item measure of risk of aberrant medication-related behavior among persons prescribed opioids for chronic pain, measured 90 days after the index E.D. visit. We used descriptive statistics and linear regressions to determine associations between satisfaction with analgesia (1–10, measured 1-day post-visit), unmet opioid preference, and opioid misuse risk by race, adjusting for age and sex.

**Results:**

We analyzed 735 participants. The mean (SD) age was 39.6 (13.6), 58.9% (*n* = 432) were female, 46.4%(*n* = 341) were White, and 36.9%(*n* = 271) were Black. Unmet preference was more common among Black (21.8%, *n* = 59) vs. White (15%, *n* = 51) participants. Black (vs White) participants had a higher median (IQR) COMM (4 (1 – 12) vs 3 (1 – 6), *P* < 0.001, and lower median satisfaction (7 (4–10) vs 8 (5 – 10), *P* = 0.002). Adjusting for unmet preference and satisfaction, Black (vs. White) participants had higher COMM (*β* = 3.4, 95% CI 1.6–5.3, *P* = 0.01). Unmet preference was associated with higher COMM (*β* = 2.3, 95% CI 1.3–3.2, *P* < 0.001). Satisfaction was associated with lower COMM (*β* = − 0.5 pp, 95% CI − 0.7, − 0.2, *P* < 0.01). In a model with a triple interaction between satisfaction, unmet preference, and race, satisfaction was associated with lower COMM (− 0.3 pp, 95% CI − 0.5, − 0.1,* P* = 0.03) and mitigated the effect of unmet preference on Black participants’ COMM (marginal effect *β* = − 1.3 pp, 95% CI − 1.9, − 0.7, *P* = 0.01).

**Conclusion:**

Satisfaction with analgesia was protective against opioid misuse risk, especially among Black participants whose opioid preference was unmet. Addressing unmet preferences and understanding factors that shape patient satisfaction with analgesia could help reduce racial disparities in opioid misuse.

**Supplementary Information:**

The online version contains supplementary material available at 10.1007/s11606-025-09637-w.

## INTRODUCTION

Racial disparities in pain treatment in the USA have been well documented. Black people are less likely to receive opioids for pain compared to similar White people across age groups and clinical settings.^1^ High rates of opioid prescribing contributed to a widely covered opioid overdose epidemic that initially affected a majority White population.^2^ Media coverage overlooked the impact of the overdose epidemic on Black people, even as overdose death rates began to rise rapidly among Black people and eventually surpass those of White people.^3^ In response to high rates of opioid overdose, there has been a reduction in opioid prescribing that has disproportionately affected Black patients with acute and chronic pain.^4^ Some media reports suggest that providers’ greater opioid vigilance with Black patients may have been protective against overdose deaths in this population.^5^ However, such reports rely on opioid prescription data, overlooking the possible impact of non-prescribed opioids on adverse opioid events. Studies show that approximately 60% of Black–White disparities in opioid prescriptions for back pain can be attributed to individual provider-level discrimination.^6^ Experiences of unfair medical treatment due to race are associated with distrust in conventional medicine and more positive attitudes toward complementary and alternative medicine.^7^ Furthermore, perceived medical discrimination is associated with greater odds of illicit drug use uniquely among Black people,^8^ and barriers to access have been documented as a motivation for the non-prescribed use of medications.^9,10^

In a previous secondary analyses of a multisite RCT of personalized opioid risk communication to E.D. patients with acute back or kidney stone pain,^11^ we found that Black patients were significantly less likely to be prescribed opioids at discharge and were more likely to experience unmet opioid preference (defined as being discharged without an opioid prescription after documented preference for opioid analgesia).^12^ We also found that personalized risk communication and receiving a prescription for opioids were both associated with decreased odds of non-prescribed opioid use 3 months later among Black participants but not among White participants.^13^

Given these findings, we hypothesized that unmet opioid preference, especially in unsatisfactory provider–patient encounters, may lead some Black patients to use opioids from nonprescribed sources.

## METHODS

### Study Design and Setting

We conducted a post hoc secondary analysis of data collected for Life STORRIED, an RCT conducted in six emergency departments at four academic medical centers.^11,14^ The study was approved by the University of Pennsylvania Institutional Review Board and pre-registered with ClinicalTrials.gov (NCT03134092). We followed CONSORT guidelines for reporting outcomes from an RCT.

### Participants

Participants were enrolled between June 2017 and August 2019. Critical inclusion criteria were (1) presentation to the E.D. for uncomplicated renal colic or musculoskeletal back or neck pain, (2) age between 18 and 70 years old, (3) English or Spanish comprehension, and (4) provider intention to discharge the patient within 24 h of enrollment. Critical exclusion criteria included any contraindication to opioids or NSAIDs and use of prescription and nonprescription opioids in the 30 days preceding E.D. presentation (see full trial protocol).^14^ Trained research associates (R.A.) identified eligible participants by reviewing the electronic medical record (EMR) and speaking with E.D. providers. Participants were surveyed daily for a week, then 14 days and 90 days after the index E.D. encounter on their pain and treatment status.

### Study Protocol and Intervention

R.A.s obtained informed consent and enrolled participants. Randomization occurred automatically through a web-based data collection platform for behaviorally oriented RCTs.^15^ After randomization, participants completed surveys that included demographics and The Opioid Risk Tool (ORT) and pain relief preference information (NSAIDs, opioids, both or neither).^16^ Additional data about the E.D. encounter were extracted from the EMR. The interventions consisted of two arms, a probability risk tool (PRT) arm and a narrative-enhanced probability risk tool (NE-PRT) arm. The PRT intervention consisted of a visual tool communicating the participants’ individualized opioid misuse risk based on the participants’ ORT score.^17^ The intervention in the NE-PRT consisted of video vignettes showing patients speaking about their experiences with acute pain and opioids in addition to the intervention used in the PRT arm. The third and control arm consisted of a standardized, general risk information sheet about opioid use (i.e., general risk comparator).

### Outcome and Covariates

The primary outcome was the current opioid misuse measure (COMM) score, which is a validated 17-item self-report measure of risk of aberrant medication-related behavior in patients receiving opioids for chronic pain.^18^ The measure’s questions are clustered into key themes: medication misuse, evidence of lying and drug use, signs/symptoms of drug misuse, emotional problems/psychiatric issues, appointment problems, and poor response to medications. This study administered the COMM questionnaire 90 days after the initial encounter.

Independent variables included sex (male vs female), age, race (White, Black, non-Black people of color), unmet opioid preference (vs all others), defined as being discharged without an opioid prescription after documented preference for opioid analgesia, and self-reported satisfaction with pain treatment, as measured the day after the initial encounter (continuous, on a scale of 0 to 10).

### Statistical Analyses

We used descriptive statistics for unadjusted analyses and performed multivariable linear regressions to determine any associations by race between pain treatment satisfaction, unmet opioid preference, and the COMM score, a measure of risk of aberrant drug behavior. In subsequent models, we introduced interaction terms between race, unmet opioid preference, and satisfaction with pain treatment (henceforth referred to as satisfaction). We clustered standard errors at the site level. All tests were two-tailed with a two-sided *p*-value of less than 0.05 for significance, and we conducted all analyses in Stata.

## RESULTS

Our final analytic sample included 735 out of 1301 participants (56.5%) who were followed to the primary endpoints of this study. This sample consisted of 341 (46.4%) White participants, 271 (36.9%) Black participants, and 123 (16.7%) NBPOC. Black participants had a median (IQR) COMM score of 4 (1–12), compared to 3 (1–6) for White participants (*P* < 0.001) and 3 (1–6) for NBPOC (*P* < 0.001). Black participants had a median (IQR) satisfaction of 7 (4–10), compared to 8 (5–10) for White participants (*P* < 0.001) and 8 (7–10) for NBPOC (*P* < 0.001) (Table 1).

**Table 1:** Participant Characteristics by Race

Characteristics	White (*n* = 341)	Black (*n* = 271)	NBPOC (*n* = 123)	*P*-value
**Sex**				**0.002**
**Male**	**150 (44.0%)**	**90 (33.2%)**	**62 (50.8%)**	
**Female**	**191 (56.0%)**	**181 (66.8%)**	**60 (49.2%)**	
**Age (SD)**	**41.4 (14.4)**	**38.0 (12.8)**	**38.4 (12.4)**	**0.005**
Satisfaction score, mean (SD)	8 (5–10)	7 (4–10)	8 (7–10)	0.01
COMM score, median (IQR)	3 (1–6)	4 (1–12)	3 (1–6)	< 0.001
COMM ≥ 9	42 (12.3%)	82 (30.2%)	23 (18.%)	< 0.001
Unmet preference	51 (15%)	59 (21.8%)	18 (14.6%)	0.06

Participants with unmet opioid reference accounted for more than 1 in 5 Black participants (*n* = 59, 21.8%), compared to 1 in 6 White participants (*n* = 51, 15%, *P* < 0.001) and 1 in 6 NBPOC (*n* = 18, 14.6%, *P* < 0.001). Participants with unmet opioid preference had lower median (IQR) satisfaction scores (7 (3–9) vs 8 (5–10), *P* < 0.001) compared to all other participants, and higher median (IQR) COMM scores (5 (2–1) vs 3 (1–6), *P* = 0.01)) (Table 2).

**Table 2:** Participant Characteristics by Unmet Opioid Preference Status

	**Control group (*n* = 607)**	**Unmet preference (*n* = 128)**	***P*-value**
**Sex**			**0.644**
Male	**247 (40.8%)**	**55 (43.0%)**	
Female	**359 (59.2%)**	**73 (57.0%)**	
**Age, mean (SD)**	**40.2 (13.5)**	**37.1 (13.5)**	**0.02**
**COMM score, median (IQR)**	3 (1–6)	5 (2–12)	< 0.001
**COMM score** ≥ **9 (%)**	105 (17.3%)	42 (32.8%)	< 0.001
**Satisfaction score, median (IQR)**	8 (5–10)	7 (3–9)	< 0.001
**Race**			0.06
White	290 (85.0%)	51 (15.0%)	
Black	212 (78.2%)	59 (21.8%)	
NBPOC	105 (85.4%)	18 (14.6%)	

In our multivariable linear regression model, Black participants had higher COMM scores than White participants (*β* = 3.3, 95% CI [1.3–5.2], *P* = 0.01). Unmet opioid preference was similarly associated with a higher COMM score (*β* = 2.2, 95% CI [1.1, 3.2],* P* = 0.004) (Table 3). Conversely, satisfaction with analgesia was associated with a lower COMM score (*β* = − 0.4 per satisfaction point, 95% CI [− 0.6, − 0.2], *P* = 0.007).

**Table 3:** Associations Between Race, Unmet Opioid Preference, Satisfaction with Pain Treatment, and Current Opioid Misuse Measure

Variable	*β* (95% CI)				
	**Model 1**	**Model 2**	**Model 3**	**Model 4**	**Model 5**
**Age**	− 0.1 (− 0.1, 0.0)	− 0.1 (− 0.1, 0.0)	− 0.1 (− 0.1, 0.0)	− 0.1 (− 0.1, 0.0)	− 0.1 (− 0.1, 0.0)
**Sex**					
Female					
Male	− 0.1 (− 1.3, 1.0)	− 0.1 (− 1.3, 1.0)	− 0.3 (− 1.8, 1.2)	− 0.2 (− 1.4, 1.1)	− 0.3 (− 1.9, 1.3)
**Race**					
White					
Black	3.3 (1.3, 5.2)	3.2 (1.1, 5.4)	2.8 (0.3, 5.3)	5.5 (0.2, 10.7)	3.6 (− 1.5, 8.8)
NBPOC	0.9 (− 1.8, 3.5)	0.9 (− 1.6, 3.4)	1.3 (− 2.4, 4.9)	0.0 (− 6.2, 6.2)	1.4 (− 6.8, 9.5)
**Satisfaction**	− 0.4 (− 0.6, − 0.2)	− 0.3 (− 0.4, − 0.3)	− 0.4 (− 0.6, − 0.2)	− 0.3 (− 0.6, − 0.1)	− 0.3 (− 0.5, − 0.1)
**Unmet preference**	2.2 (1.1, 3.2)	5.2 (− 0.0, 10.4)	1.5 (− 0.4, 3.5)	2.1 (1.0, 3.2)	2.7 (− 3.0, 8.5)
**Unmet preference # satisfaction**		− 0.5 (− 1.2, 0.2)			− 0.2 (− 1.3, 0.9)
Race # satisfaction					
Black # satisfaction				− 0.3 (− 1.0, 0.3)	− 0.1 (− 0.8, 0.6)
NBPOC # satisfaction				0.1 (− 0.5, 0.7)	− 0.0 (− 0.7, 0.6)
**Race #unmet preference**					
Black # unmet preference			2.4 (− 3.4, 8.3)		5.5 (1.8, 9.2)
NBPOC # unmet preference			− 2.7 (− 10.1, 4.6)		− 5.8 (− 10.7, − 0.9)
**Race # unmet preference #satisfaction**					
Black # unmet preference # satisfaction					− 0.7 (− 2.0, 0.8)
NBPOC # unmet preference # Satisfaction					0.5 (− 0.4, 1.4)

**Figure 1:**
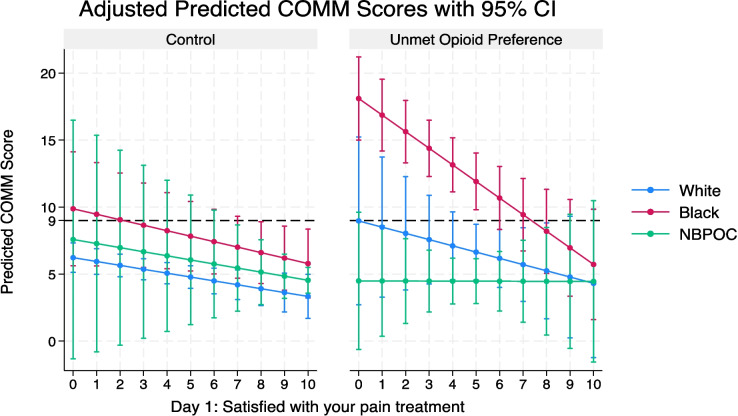
Association between pain treatment satisfaction and current opioid misuse measure scores by race and unmet opioid preference status.

In a model with interaction terms between race, satisfaction, and unmet opioid preference, greater satisfaction remained independently associated with lower COMM scores (*β* = − 0.3 per satisfaction point, 95% CI − 0.5 to − 0.1, *P* = 0.03). The effect of unmet preference on greater COMM was significant for Black (vs White) participants (*β* = 5.5, 95% CI 1.8–9.2, *P* = 0.02), but was mitigated significantly by satisfaction (see Fig. 1).

**Figure 2:**
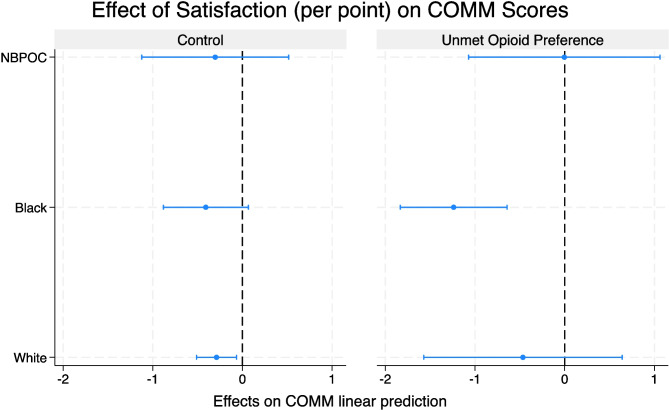
Per point marginal effect of satisfaction with pain treatment on current opioid misuse measure (COMM) by race and unmet opioid preference.

Using the margins function, we found the effect of satisfaction on reducing COMM scores to be statistically significant for White participants who did not experience unmet opioid preference (*β* = − 0.3 per satisfaction point, 95% CI [− 0.5, − 0.1], *P* = 0.03), and Black participants who experienced unmet opioid preference (*β* = − 1.2 per satisfaction point, 95% CI [− 1.8, − 0.6],* P* = 0.01). In other words, the effect size of satisfaction on COMM scores among Black participants who experienced unmet opioid preference was over 4 times the effect size among White participants who did not experience unmet opioid preference (see Fig. 2).

To account for the fact that prescribers may avoid prescribing opioids to patients with high ORT scores given their increased risk of opioid misuse, we performed secondary analyses adjusting for participants’ ORT scores. The results of this analysis were similar and remained statistically significant (Appendix 1).

## DISCUSSION

Our study has three main findings. First, among patients presenting to the ED with acute back or kidney stone pain, Black patients had greater opioid misuse risk 90 days after the initial encounter based on a previously validated measure. Second, unmet opioid preference was significantly associated with increased opioid misuse risk, but only among Black participants. Third, satisfaction with pain treatment was independently associated with lower opioid misuse risk, with the most potent effect on Black participants who experienced unmet opioid preference.

The finding that unmet opioid preference had a unique effect on opioid misuse risk among Black participants is consistent with our prior analyses of this cohort, in which we found that receiving a prescription for opioids at discharge was associated with lower odds of reported non-prescribed opioid use.^13^ Non-prescribed opioid use is a unidimensional measure, and confirming the effect of unmet opioid preference using the COMM score, a validated, multidimensional measure, is a novel contribution to the literature.

Similarly novel to our study is the finding that satisfaction with pain treatment significantly mediates the impact of unmet preference on opioid misuse, especially among Black participants. A few potential explanations may underlie this finding. While unmet preference may lead some individuals to seek relief through nonprescribed opioids, various factors during a clinical encounter may mitigate such risks, such as the expression of empathy, patient-centered communication, and patient education about effective therapies and opioid risk. Prior studies have found that physicians use less patient-centered language and show a lower level of positive affect with Black patients compared to White patients.^19^ These aspects of a clinical encounter can, in turn, be interpreted by patients negatively, and impact their satisfaction and follow-through with adherence to guidance including safe medication use.

It is possible that Black patients’ pain was underappreciated and thus undertreated in the acute setting, leading those with unmet preferences to seek relief via non-prescribed opioids. Evidence suggests that undertreated acute pain increases patients’ risk of developing chronic pain and persistent opioid use. Additionally, other studies have found, although different from “unmet preference,” that perceived discrimination in medical encounters is associated with greater odds of reporting illicit drug use.^8,20^ Perceived discrimination in medical settings has also been associated with more distrust in conventional medical settings and more favorable attitudes toward complementary or alternative medicine among Black people.^7^ For example, in the treatment of opioid use disorder, the use of non-prescribed buprenorphine to treat opioid use disorder was due to unmet needs and preferences from the care delivery system.^21,22^

Our findings have multiple implications. First, unmet opioid preference in the acute setting may be an overlooked pathway through which some Black people become vulnerable to opioid misuse. This is especially concerning given the evolution of the opioid overdose epidemic^2,23^ and increasingly unsafe opioid supply.^24^ Second, aspects of clinical encounters that shape patients’ satisfaction with their pain treatment may be among the tools to leverage to reduce adverse opioid events, especially among Black patients. Further studies should seek to understand factors that shape patients’ satisfaction with pain treatment and their potential impact on opioid misuse and adverse opioid events.

The racial and geographic diversity in our sample is a strength, as is the longitudinal nature of the study. We used satisfaction with pain treatment measured the day after the encounter, expecting the most accurate recall, and administered the COMM 3 months after the encounter, not simultaneously, which strengthens the argument for a causal relation between satisfaction with pain treatment and opioid misuse. Limitations of this study include the use of the COMM score, which was validated in a non-representative sample (83% White, 61% women, and 87.3% high school graduates).^25^ As a result, the assessment of opioid misuse risk may not be as valid an assessor of aberrant drug behavior in the racially diverse sample used in this study. However, we have previously found similar results using single metric measures of opioid misuse, such as non-prescribed opioid use. Participants in this study were followed for only 3 months with significant attrition, and opioid misuse risk as an outcome may not be as clinically significant over this timeline. A longer-term follow-up with this cohort would be necessary to confirm the clinical significance of our findings.

## CONCLUSION

In this secondary analysis of an RCT of personalized opioid risk communication among E.D. patients with acute low back or kidney stone pain, we found that unmet opioid preference was uniquely associated with greater opioid misuse risk among Black patients. Additionally, satisfaction with pain treatment following the encounter was independently associated with lower opioid misuse risk and a reduction of the effect of unmet opioid preference on opioid misuse risk, especially among Black participants. Undertreated pain and unmet patient preferences for analgesia may be an overlooked pathway through which Black people become more vulnerable to opioid misuse and associated opioid adverse events, especially in the context of the US opioid overdose epidemic. Future work should explore factors that shape patient satisfaction as a potential way to mitigate opioid-related risk and reduce racial disparities in opioid adverse events.

## Supplementary Information

Below is the link to the electronic supplementary material.

Supplementary file1 (DOCX 105 KB)

## Data Availability

Data strictly specific to this manuscript may be available upon request.
