# Supplementary material for: Racial Disparities in Unmet Pain Treatment Preference, Pain Treatment Satisfaction and Subsequent Opioid Misuse: A Secondary Analysis of a National Multisite RCT
Source: J Gen Intern Med. 2025 Jun 25;41(4):1041–7. doi: 10.1007/s11606-025-09637-w (PMC13009308; doi:10.1007/s11606-025-09637-w)
Supplement: Supplementary file 1 — Supplementary file1 (DOCX 105 KB) [file 11606_2025_9637_MOESM1_ESM.docx]

**Appendix 1:** Association between pain treatment satisfaction and current opioid misuse measure scores by race and unmet opioid preference status**,** adjusting for age, sex and opioid risk tool score
